# Editorial: Insights and regulation of plant carbon metabolism

**DOI:** 10.3389/fpls.2022.1011224

**Published:** 2022-08-31

**Authors:** Rubén Vicente, Maria Grazia Annunziata, Diana Santelia

**Affiliations:** ^1^Plant Ecophysiology and Metabolism Group, Instituto de Tecnologia Química e Biológica António Xavier (ITQB NOVA), Universidade Nova de Lisboa, Oeiras, Portugal; ^2^Department of Molecular Biology, Institute of Biochemistry and Biology, University of Potsdam, Potsdam, Germany; ^3^Institute of Integrative Biology, ETH Zurich, Zurich, Switzerland

**Keywords:** carbohydrates, crop production, fluctuating environment, metabolic regulation, phenotyping, photosynthesis, plant carbon metabolism, source-to-sink dynamics

In their natural environments, plants use sunlight to obtain the energy needed to carry out the fixation of atmospheric CO_2_ (photosynthesis) and other anabolic processes that are necessary for their growth and development. This energy is also used for the daily challenge of survival during the night, when the absence of light forces the plant to use the carbon reserves accumulated during the day. In addition to these daily fluctuations, plants efficiently regulate their carbon metabolism at the whole plant level to adapt to changes in environmental conditions. During the day, the autotrophic organs (“sources”) supply carbon and energy, mainly in the form of sucrose, to maintain plant metabolism and ensure the development of heterotrophic organs (“sinks”), such as roots and reproductive structures. When carbon fixation exceeds its demand, carbon is accumulated in transient carbon pools (mainly starch and soluble sugars) to be used during the night or at later growth stages. Carbon metabolism is highly susceptible to environmental changes, being involved in stress sensing and signaling that allow plants to adapt to the new growth conditions. Moreover, efficient source-to-sink transport of sugars is essential for plant growth processes and enables fine-tuned carbon partitioning across plant organs through the phloem. Despite huge progress over the last decades in understanding plant carbon metabolism, knowledge about its regulation and coordination at the whole-plant level is still fragmentary.

This Research Topic aims at providing novel insights into the regulation of plant carbon metabolism. Matiolli et al. studied one of the key mechanisms regulating carbon metabolism: the post-translational modifications (PTMs) of proteins involved in primary metabolism. The authors provided a holistic overview of PTMs in plant carbon metabolism, showing how they can regulate carbon sensing, fixation, storage, remobilization, transport, and cytosolic glycolysis. PTMs lead to fast, often reversible, adjustments of processes such as protein-protein interactions, enzymatic activities, stability and subcellular localization, which allow to optimize plant fitness in the ever-changing environment. Among the most relevant examples, the role of three conserved kinases (SnRK1, TOR, and HXK1) and the ferredoxin/thioredoxin system controlling carbon-energy-nutrient homeostasis were highlighted. Furthermore, Paul reviewed one of the most controversial points about carbon metabolism over the past years: is photosynthesis one of the main targets to focus on to improve crop yields? While some studies suggest that photosynthesis is not the main limiting process for crop improvement, other studies have shown that its manipulation led to significant improvements. However, the improvement of the plant carbon status has to be accompanied by other crucial factors, such as optimized nitrogen and water supplies and source-sink interactions. Martínez-Peña et al. studied source-sink dynamics in field-grown durum wheat (*Triticum turgidum* L. ssp. *durum*) by integrating canopy phenotyping with metabolic analyses of different plant photosynthetic organs. This novel approach allowed them to investigate the coordination between carbon and nitrogen metabolism at the whole plant level. Interestingly, the authors observed that non-foliar organs (e.g. ear organs such as awns, glumes, and lemmas) play a crucial role in late grain filling and grain quality, although their metabolic activity is quantitatively not comparable to flag leaves. These findings lead to the suggestion that non-foliar organs may represent novel targets for crop breeding.

Photoassimilates are the main source of carbon and energy in sink organs that are transported from sources by various mechanisms. The transport of sugars through the phloem requires the subsequent unloading via sugars transporters, as highlighted in the study of Li et al. The authors revealed the role of the hexose transporter CsSWEET7a, localized in the phloem region of the receptacle and nectary in both male and female flowers of cucumber (*Cucumis sativus* L.), in supplying sugars for flower anthesis and nectar secretion to reward pollinators. Continuing with the reproductive phase of flowering plants, Wang et al. further showed the involvement of carbon metabolism in pollen germination in *Arabidopsis thaliana*. This study reveals that starch and sucrose metabolism were induced during the transition from mature to hydrated pollen, while the metabolites palmitic acid, oleic acid, linolenic acid, quercetin, luteolin/kaempferol, and γ-aminobutyric acid (GABA) may contribute to activate pollen tube emergence. Hence, sugars play an essential role in plant fertilization, seed formation, and yield. Photoassimilates also play a primary role in root growth, as Kang et al. demonstrated in radish (*Raphanus sativus* L.) with the levels of fructose determining root length and color. Glucose was the main sugar in radish roots, while sucrose was majorly transported to roots by the apoplasmic pathway, where it was metabolized. Sucrose and cellulose synthases were correlated at transcript levels, suggesting tightly coordinated cell wall synthesis, while the excess fructose generated by sucrose synthases was predominantly phosphorylated by fructokinases.

New findings on the reserve polysaccharide starch are reported by Ribeiro et al. which highlights its central role in plant responses to biotic and abiotic stresses. Starch synthesis is generally decreased under abiotic stresses (e.g., salt, drought, heat, and cold stresses) due to lower photosynthetic rates, while its content also decreases to reallocate carbon or to synthesize osmo/cryoprotectants to minimize the impact of stress. In response to pathogen infection, starch usually accumulates, which may restrict CO_2_ diffusion. However, the dynamics of starch metabolism in response to stress is dependent on several factors, and further research studies should consider carefully the strength and duration of the stress, and short- (daily fluctuations regulated by circadian clock) and long-term responses. In line with this, Calderan-Rodrigues et al. reports novel findings on the role of proteogenic dipeptides in the control of metabolic fluxes during the day-night cycle of *Arabidopsis thaliana*. Intriguingly, they found rhythmic dipeptides under short- and long-day diel cycles that could be regulated by the Target of Rapamycin (TOR) signaling pathway. The authors hypothesized that altered levels of proline and proline-containing didpeptides (e.g., Pro-Gln) under limited carbon availability may act as alternative respiratory substrates to allow plant survival and modulate protein activity. External application of metabolic compounds is also a direct way to improve plant growth and stress response. Abd Elbar et al. applied GABA to leaves of tomato (*Solanum lycopersicum* L.) plants under chilling stress at the reproductive and fruit set stages. This non-proteinogenic amino acid, known to be associated with stress tolerance, led to fruit yield improvement under such stress. This response was mainly associated with an upregulation of sugar metabolism (acting as osmolytes), modulation of pigment composition and secondary metabolism, alleviation of oxidative damage by promoting antioxidant system and maintenance of the integrity of plastids' ultrastructure.

## Concluding remarks

The regulation of carbon metabolism is key to the understanding of how plants use their internal reserves to respond to the environment and develop a specific phenotype, which ultimately has an essential impact on plant growth, development and yield. Over the past decades, much research has been carried out to understand the mechanisms regulating carbon fixation and its utilization in model plants, mainly *Arabidopsis thaliana*. However, the knowledge gained studying model species is more and more applied to crops of economic or environmental interest and, in addition, researchers have understood the importance of conducting experiments under conditions as close as possible to the real growing environments. In this Research Topic and throughout the aforementioned studies, we highlight the need to advance the study of mechanisms regulating carbon metabolism as the backbone for crop improvement. Holistic studies should be promoted that (i) integrate approaches at different levels, from molecular and biochemical methods to high-precision phenotyping techniques, (ii) encompass the whole plant (source-sink dynamics) at different stages of its development, and (iii) include the different crops of interest under present and future growth conditions.

## Author contributions

RV wrote the manuscript, while MA and DS revised and edited it. All authors approved the submitted version for publication.

## Funding

RV acknowledges the support by FCT - Fundação para a Ciência e a Tecnologia, I.P., through GREEN-IT - Bioresources for Sustainability R&D Unit (UIDB/04551/2020, UIDP/04551/2020) and LS4FUTURE Associated Laboratory (LA/P/0087/2020). MA acknowledges funding by the Deutsche Forschungsgemeinschaft (DFG) of the Collaborative Research Center 973 Priming and Memory of Organismic Responses to Stress (www.sfb973.de) and by the European Union's Horizon 2020 Research and Innovation Programme, project PlantaSYST (SGA-CSA No. 739582 under FPA No. 664620). DS acknowledges the support by Swiss National Science Foundation (Grant no. 310030_185241) and by ETH Zurich.

## Conflict of interest

The authors declare that the research was conducted in the absence of any commercial or financial relationships that could be construed as a potential conflict of interest.

## Publisher's note

All claims expressed in this article are solely those of the authors and do not necessarily represent those of their affiliated organizations, or those of the publisher, the editors and the reviewers. Any product that may be evaluated in this article, or claim that may be made by its manufacturer, is not guaranteed or endorsed by the publisher.

